# Altered Brain Functional Connectivity in Female Athletes Over the Course of a Season of Collision or Contact Sports

**DOI:** 10.1089/neur.2022.0010

**Published:** 2022-09-19

**Authors:** Alyssia Wilson, W. Dale Stevens, Lauren Sergio, Magdalena Wojtowicz

**Affiliations:** ^1^ Department of Psychology, York University, Toronto, Ontario, Canada; ^2^ School of Kinesiology, York University, Toronto, Ontario, Canada

**Keywords:** contact sports, female athlete, functional connectivity, repetitive head impact

## Abstract

University athletes are exposed to numerous impacts to the body and head, though the potential cumulative effects of such hits remain elusive. This study examined resting-state functional connectivity (rsFC) of brain networks in female varsity athletes over the course of a season. Nineteen female university athletes involved in collision (*N* = 12) and contact (*N* = 7) sports underwent functional magnetic resonance imaging scans at both pre- and post-season. A group-level independent component analysis (ICA) was used to investigate differences in rsFC over the course of a season and differences between contact and collision sport athletes. Decreased rsFC was observed over the course of the season between the default mode network (DMN) and regions in the frontal, parietal, and occipital lobe (*p* false discovery rate, ≤0.05) driven by differences in the contact group. There was also a main effect of group in the dorsal attention network (DAN) driven by differences between contact and collision groups at pre-season. Differences identified over the course of a season of play indicate largely decreased rsFC within the DMN, and level of contact was associated with differences in rsFC of the DAN. The association between exposure to repetitive head impacts (RHIs) and observed changes in network rsFC supplements the growing literature suggesting that even non-concussed athletes may be at risk for changes in brain functioning. However, the complexity of examining the direct effects of RHIs highlights the need to consider multiple factors, including mental health and sport-specific training and expertise, that may potentially be associated with neural changes.

## Introduction

Contact and collision sport athletes are exposed to repetitive head impacts (RHIs) attributable to rapid changes in head velocity as the result of a direct contact to the head or collision to the body.^1^ These impacts often lack visible signs of injury and/or may result in subthreshold clinical symptoms in comparison with hits resulting in concussion(s).^2^ However, there is a growing literature examining potential cumulative effects of RHIs on athletes' physical, cognitive, and emotional health.^3–6^ To date, studies examining the possible cognitive effects of RHIs have revealed mixed findings. Several studies have identified decreases in neurocognitive performance over the course of an athletic season associated with sport exposure in the absence of reported concussions.^4,5,7–12^ However, collision, contact, and non-contact athletes are a heterogenous group who have been shown to experience variable neurological outcomes based on their relative exposure to RHIs.^13^

The available research examining evidence of differences in structural brain metrics associated with RHIs has also reported mixed findings. Some studies have not observed white matter or cortical volume differences over the course of a season in collision athletes.^14,15^ However, others have reported changes in multiple structural and neurometabolic metrics across short-term^16–18^ and long-term findings,^19^ as well as differences between collision and non-contact athletes at baseline.^17,18^

Using resting-state functional magnetic resonance imaging (rsfMRI), several studies have identified changes to resting-state functional connectivity (rsFC) after exposure to RHIs.^4,8,15,20–25^ Across brain regions, rsFC may detect subtle changes in the neural integrity of brain regions and networks that may be vulnerable to head impacts.^26,27^ Research on male football athletes has revealed differences in rsFC among asymptomatic football players over the course of a season.^8,15,21,22^ These studies have consistently observed changes associated with default mode network^21,22^ (DMN) connectivity.^15^ Collegiate rugby players showed connectivity differences after a single game between frontal, parietal, and subcortical regions.^24^ In male soccer players, rsFC increases in the central autonomic network have been observed in association with the amount of head-to-ball impact exposure over a season.^25^ Difference in global neural connectivity have been observed in varsity athletes at baseline, with the highest global connectivity observed in non-contact athletes and lowest global connectivity observed in collision athletes, suggesting potential better functional neural integrity in lower contact athletes.^23^

The majority of the literature examining RHIs has focused on male football players. Of the total number of imaging studies examining RHIs (*N* = 12), only three included both male and female athletes, whereas just one study included female athletes only. See the Supplementary Material for an in-depth literature review. Despite accounting for 43% of the collegiate athlete population,^28^ female athletes have been severely understudied in this literature. The type of impacts that female athletes sustain may be different than their male counterparts, attributable, in part, to smaller neck size and differences in type of play.^29–33^ In addition, some studies report that females experience more adverse symptoms,^34,35^ greater symptoms of depression,^36^ and worse neurocognitive performance.^16,34,37,38^ This suggests potential sex-related differences in outcomes after RHIs. Few studies have examined neural differences in female athletes associated with RHI; however, two studies have shown structural brain changes in female hockey and rugby players that are not observed in male players.^16,20^

This study examined cognitive and neural functioning of a cohort of female varsity athletes over the course of a season of play. Given the broad range of symptom presentations in female and male athletes after a head injury,^34,37–40^ and the previous focus in the literature on DMN findings, we decided to expand the scope of our study by taking a broader whole-brain network approach. Specifically, we examined changes in rsFC within the DMN, sensorimotor (SMN), frontoparietal (FPN), dorsal attention (DAN), and salience (SN) networks post-season compared to pre-season in female athletes involved in collision and contact sports. These networks have been implicated in head injury,^24,41–43^ motor function,^44,45^ executive functions,^46–48^ and attention processes,^48–51^ respectively. Potential rsFC differences in these networks associated with level of contact (i.e., collision vs. contact) were also explored.

This study hypothesized that: 1) there would be differences in rsFC between contact and collision athletes in the DMN and 2) a difference in rsFC would be observed over the course of a season. To further inform the literature, we also planned exploratory analyses in the following networks: SMN, FPN, DAN, and SN. A better understanding of rsFC differences in athletes over a season of play will improve our knowledge of the possible neurobiological changes associated with subclinical head impact that may compound over time and put exposed players at greater risk of clinical injury.

## Methods

### Participants

Nineteen female university athletes (ages 18–21) were recruited from York University. Participants were eligible if: 1) they were >18 years of age, 2) were rostered to play on a university team for the current season, and 3) met the requirements for safe magnetic resonance imaging (MRI) scanning. Twelve were involved in collision sport (i.e., ice hockey), whereas the remainder (*n* = 7) were involved in contact sports (i.e., basketball, soccer, or volleyball). Level of contact was determined in accordance with the classifications proposed by Rice,^52^ determined by estimating the level of relative risk for an acute injury. Collision sport athletes often come into rough contact with other players or objects usually at higher speeds, whereas contact sport athletes often come into contact with each other as a by-product of the sport and with relatively less force.

### Procedure

This study was approved by the Human Participants Review Sub-Committee of York University's Ethics Review Board. Participants provided written informed consent and completed testing and MRI scanning before the start of their athletic season (before beginning contact practices) and immediately after their athletic season. Scans took place, on average, 166 days (standard deviation [SD] = 45) apart.

### Measures

#### Sport Concussion Assessment Tool 5

The Sport Concussion Assessment Tool 5 (SCAT5)^53^ was used to assess concussion symptoms by using scores from the Standardized Assessment of Concussion (SAC) and the total number of symptoms from the Symptom Evaluation.

#### Attention Network Test-Interaction

The Attention Network Test-Interaction (ANT-I) is a brief task of attention task and executive control that uses 2 (alerting) × 3 (orienting) × 2 (executive) design. It is described in detail elsewhere^41^ and has been previously used in concussion research.^55–58^ Briefly, efficiency of the alerting network is examined by the difference in reaction time on trials presented with a tone versus no tone. Efficiency of orienting is examined by differences in reaction time on trials with an invalid spatial cue (i.e., asterisk that appears in the opposite location of the target stimuli) versus a valid spatial cue. Finally, the efficiency of the executive network is examined by the difference in reaction time on incongruent flanker trials (i.e., the target arrow is surrounded by arrows pointing in the opposite direction) versus congruent trials (i.e., all arrows pointing in the same direction).

#### Magnetic resonance imaging acquisition

MRI data were acquired using a 3 Tesla Siemens TRIO MRI scanner (Siemens, Erlangen, Germany). Images were acquired for each subject before the beginning of the season and after the season. Whole-brain, high-resolution, T1-weighted anatomical scans were collected using an ascending multi-slice three-dimensional magnetization-prepared rapid acquisition gradient echo sequence (field of view [FOV] = 256 mm, 1.0 × 1.0 × 1.0 mm voxels, repetition time [TR] = 2300, echo time [TE] = 2.62 ms). A whole-brain multi-echo planar imaging sequence (T2*-weighted) was used to acquire functional data in 240 volumes (43 slices, FOV = 216 mm, 64 × 64 matrix, 3.4 × 3.4 × 3.0 mm^3^ voxels, TR = 3000 ms, TEs = 14 ms, 30 ms, 46 ms, flip angle = 83 degrees). During rsfMRI scanning, patients were instructed to lay still with their eyes closed, but not to fall asleep.

### Functional imaging pre-processing

rsfMRI data were pre-processed using the CONN toolbox Version 18.b(59) on the Matlab Version R2019a platform (The MathWorks, Inc., Natick, MA). The default pre-processing pipeline was used, and functional data were functionally realigned and unwarped, translated by centering to (0,0,0) coordinates, slice-time corrected, scrubbed with Artifact Detection Tools–based identification for outlier scans, segmented into gray matter, white matter, and cerebrospinal fluid, normalized to the Montreal Neurological Institute (MNI) template MNI152, and smoothed using an 8-mm Gaussian kernel, full width at half maximum. Denoising was performed using the toolbox's aCompCor protocol that involved linear regression of nuisance parameters to reduce physiological and movement effects. Finally, data were linearly detrended and band-pass filtered (0.008–0.090 Hz).

### Independent component analysis

**FIG. 1.**
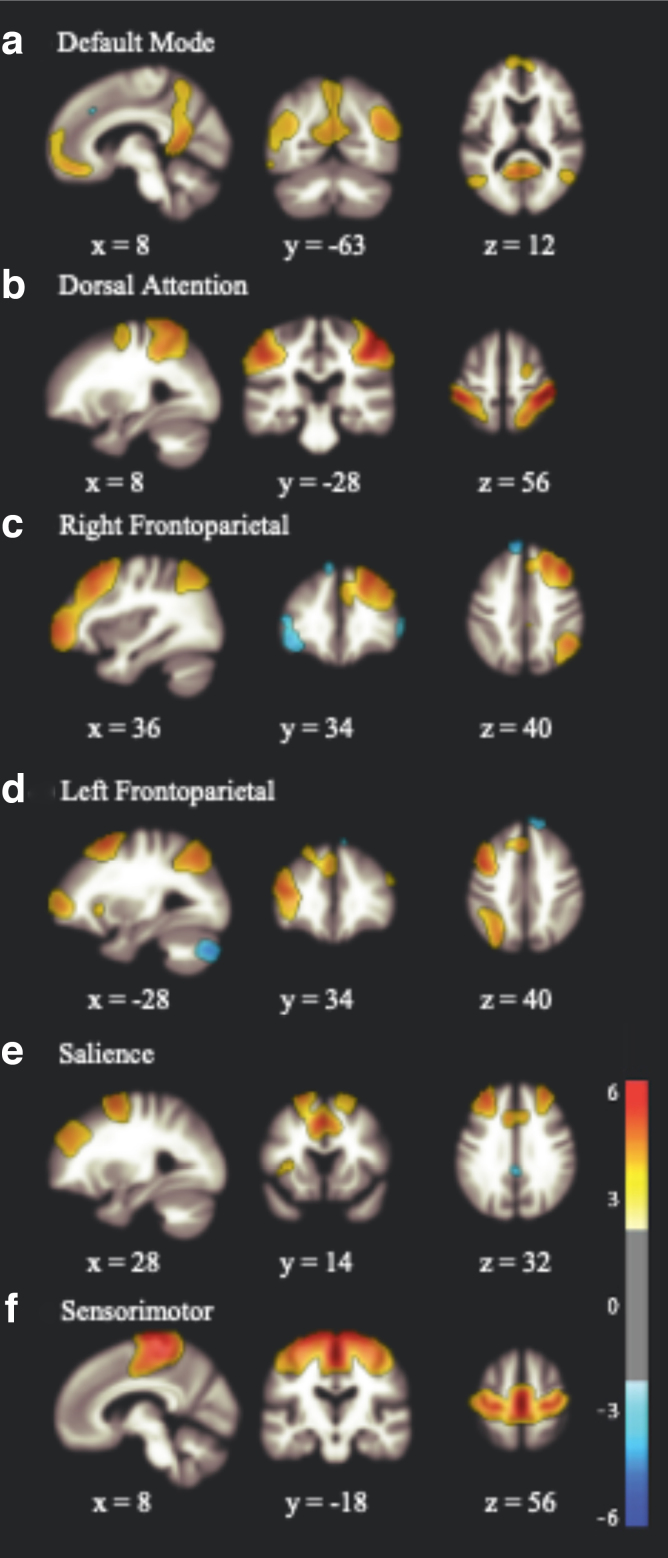
Results of ICA showing components with high match to template for the (**A**) DMN, (**B**) DAN, (**C**) left FPN, (**D**) right FPN, (**E**) SN, and (**F**) SMN. DAN, dorsal attention network; DMN, default mode network; FPN, frontoparietal network; ICA, independent component analysis; SMN, sensorimotor network; SN, salience network.

Using a whole-brain, multi-variate approach, rsFC data were analyzed with the independent component analysis (ICA) pipeline in CONN toolbox.^59^ FastICA was used to estimate 20 independent spatial components. Group independent analysis setting was used to back-project individual subject-level spatial map estimation. and the dimensionality reduction value was 64. Spatial match-to-template correlations between each group-level spatial map and the CONN toolbox pre-defined networks, based on Harvard-Oxford and Automated Anatomical Labeling atlases, were used to identify resting-state networks. Specific networks were chosen based on involvement in clinical symptoms associated with RHIs, including the DMN, SMN, FPN, DAN, and SN (see Fig. 1).

### Statistical analysis

#### Behavioral variables

Because normality was found to be violated on some measures, non-parametric tests were used where relevant. Mann-Whitney U tests were used to compare mean rank of age, age at first sport, concussion history (number of previous concussions) between groups at pre-season and SCAT total number of symptoms, and SAC subtotal between groups at pre- and post-season. Wilcoxon's signed-rank tests were run between pre- and post-season scores to compare mean rank for all athletes assessed at both time points.

For the ANT-I, mean reaction times and intraindividual variability using the individual standard deviations^54^ (ISDs) were calculated for each participant across trials and were both compared in separate repeated-measures analysis of variance (ANOVA). Twelve condition scores were calculated, one for every combination of alerting (tone vs. no tone), orienting (valid, invalid, or no cue), and executive control (congruent vs. incongruent flankers) factors, which were compared across group (level of contact) and time (pre- vs post-season) in a 2 × 3 × 2 × 2 × 2 repeated-measures ANOVA. Repeated-measure ANOVAs for group and time were performed for ISDs. All analyses were adjusted for multiple comparisons.

### Resting-state functional magnetic resonance imaging analysis

A 2 × 2 ANOVA was used to assess the main effects of group (contact and collision) and time (pre- and post-season), as well as any group × time interaction. We performed follow-up analyses for the main effect of time on the rsFC of significant ICA components in each group independently. We also performed a follow-up analysis for the main effect of group at each time point independently, using between-subject *t*-tests. Non-parametric tests were used with an uncorrected voxel threshold of *p* ≤ 0.001 and a cluster-mass correction of *p* ≤ 0.05 false discovery rate (FDR) to control for multiple comparisons.

## Results

### Participant characteristics

At pre-season, there were no significant differences in age, age at first sport, concussion history, or SAC scores between contact and collision athletes (*p*s > 0.05; see Table 1). Contact athletes, however, did have significantly higher total SCAT5 symptom scores at preseason (see Table 1). There were no significant differences in scores between pre- and post-season scores (*p*s > 0.05; see Table 1). No athletes sustained a concussion over the course of the season.

**Table 1.** Demographic and Clinical Results for Contact and Collision Athletes

	Pre-season			Post-season		
	Contact (*n* = 7)	Collision (*n* = 12)	*p*(*g*)	Contact (*n* = 7)	Collision (*n* = 12)	*p*(*g*)
Age, years, M (SD)	19.71 (1.38)	19.50 (1.09)	0.65 (0.17)	—	—	—
Age at first sport, M (SD)	7.28 (3.68)	4.58 (0.90)	0.08 (1.12)	—	—	—
Concussion history, M (SD)	1.57 (1.51)	0.58 (0.90)	0.14 (0.82)	—	—	—
SCAT5 total symptoms, M (SD)	4.71 (3.40)	1.33 (3.11)	0.02 (1.00)	4.14 (4.60)	2.00 (3.22)	0.34 (0.54)
SAC subtotal, M (SD)	27.43 (1.81)	26.83 (1.19)	0.43 (0.39)	26.86 (0.90)	27.92 (1.56)	0.17 (0.74)

*g* = Hedge's *g*.

M, mean; SD, standard deviation; SCAT5, Sport Concussion Assessment Tool 5; SAC, Standardized Assessment of Concussion.

### Attention Network Test-Interaction results

ANT-I results are presented in Table 2. Overall scores showed within-subject effects of the alerting, orienting, and executive function networks (Alerting: *F*_(1,17)_ = 5.66, *p* = 0.029; Orienting: *F*_(2,34)_ = 86.03, *p* < 0.001; Executive: *F*_(1,17)_ = 527.96, *p* < 0.001). *Post hoc* analyses revealed that participants responded more quickly when there was a tone, valid cue, or congruent flanker in the alert, orient, and executive conditions, respectively (*p*s ≤ 0.05).

**Table 2.** Attention Network Test-Interaction Results

	Pre-season		Post-season	
	Contact (*n* = 7)	Collision (*n* = 12)	Contact (*n* = 7)	Collision (*n* = 12)
Alerting				
Tone, M (SD)	617.11 (79.29)	580.21 (56.29)	596.92 (60.67)	543.35 (40.10)
No tone, M (SD)	618.44 (128.88)	580.83 (116.93)	606.97 (59.36)	549.76 (42.03)
Orienting				
Valid cue, M (SD)	573.47 (81.32)	539.84 (49.88)	559.18 (53.23)	508.12 (29.60)
Invalid cue, M (SD)	635.39 (80.11)	597.07 (63.64)	613.70 (64.91)	559.09 (51.00)
No cue, M (SD)	644.46 (77.57)	604.66 (60.75)	632.96 (64.85)	572.46 (50.03)
Executive Control				
Congruent, M (SD)	575.34 (75.83)	540.10 (52.00)	561.36 (57.98)	512.25 (37.20)
Incongruent, M (SD)	660.21 (81.10)	620.95 (61.41)	642.53 (62.35)	580.86 (44.76)

M, mean; SD, standard deviation.

There was also a significant main effect of time (*F*_(1,17)_ = 6.91, *p* = 0.018; partial eta squared = 0.29), with faster performance observed at post-season compared to pre-season (M_pre_ = 599.15 vs. M_post_ = 574.25). ANT-I performance did not differ by group (*F*_(1,17)_ = 2.66, *p* = 0.121, partial eta squared = 0.14), and there was no group by time interaction (*F*_(1,17)_ = 0.917, *p* = 0.352, partial eta squared = 0.05), indicating no group differences in pre- and post-season testing (see Table 2 for mean (M) and SDs of ANT-I results by group at the two time points).

A repeated-measures ANOVA of ISDs did not show significant effects of time (*F*_(1,17)_ = 2.47, *p* = 0.625, partial eta squared = 0.014) or group (*F*_(1,17)_ = 1.297, *p* = 0.270, partial eta squared = 0.071).

### Resting-state functional connectivity results

**FIG. 2.**
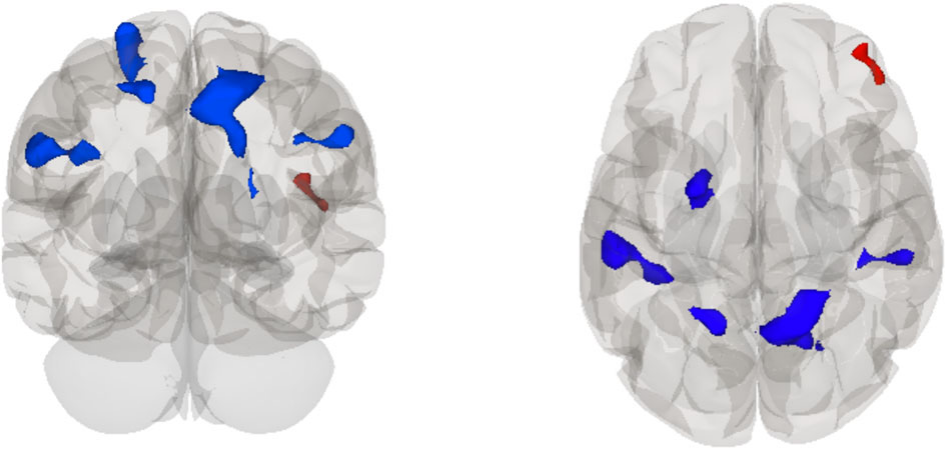
Glass brain rendering showing the independent component analysis (ICA) main effect of time on rsFC of the default mode network (DMN): decreased connectivity with the right superior parietal lobule, left superior frontal gyrus, bilateral supramarginal gyrus, left superior parietal lobule, and increased connectivity with the right frontal pole at post-season compared to pre-season. rsFC, resting-state functional connectivity.

There was a main effect of time, such that the DMN (see Fig. 2) had decreased rsFC at post-season with a relatively large cluster primarily in the superior parietal lobule, with some voxels in the superior division of the lateral occipital cortex and spreading into the pre-cuneus and post-central gyrus in the right hemisphere (*T* = −6.85, pFDR = 0.001) and a cluster across the supramarginal gyri and post-central gyrus bilaterally (left, *T* = −6.13, pFDR = 0.004; right, *T* = −6.41, pFDR = 0.043). Another cluster primarily in the left superior frontal gyrus with spread into the pre-central gyrus (*T* = −7.61, pFDR = 0.006) and one primarily in the superior parietal gyrus and lateral occipital cortex (*T* = −5.82, pFDR = 0.043) also showed decreased rsFC at post-season. Increased rsFC with the DMN was observed with a cluster in the right frontal pole (*T* = 6.27, pFDR = 0.043) at post-season.

A follow-up within-subjects *t*-test analysis of the contact group only showed significantly decreased rsFC between the DMN and three clusters bilaterally in the lateral occipital cortex (right, *T* = −19.32, pFDR = 0.001; right, *T* = −10.18, pFDR = 0.003; left, *T* = −16.42, pFDR = 0.003), left supramarginal gyrus (*T* = −27.85, pFDR = 0.001), left superior frontal gyrus (*T* = −13.75, pFDR = 0.003), bilateral superior parietal lobules (left, *T* = −12.81, pFDR = 0.010; right, *T* = −11.03, pFDR = 0.045), and right pre-central gyrus (*T* = −10.08, pFDR = 0.045) at post- versus pre-season. No significant rsFC differences were observed between the DMN and other brain regions in a within-subjects *t*-test of the collision group at pre- versus post-season (see Table 3).

**Table 3.** ICA Results

		Cluster size	MNI coordinates			T value	Mass pFDR
Network	Primary regions		x	y	z		
Main effect of time (*n* = 19)							
DMN	Superior parietal lobule right	645	18	–46	70	–6.85	0.001
	Supramarginal gyrus, anterior division left	357	–50	–26	32	–6.13	0.004
	Superior frontal gyrus left	268	–22	–4	70	–7.61	0.006
	Supramarginal gyrus, anterior division right	145	56	–32	40	–6.41	0.043
	Superior parietal lobule left	133	–16	–58	68	–5.82	0.043
	Frontal pole right	115	42	48	–16	6.27	0.043
Follow-up: contact (*n* = 7) Pre(−1) Post(1)							

**FIG. 3.**
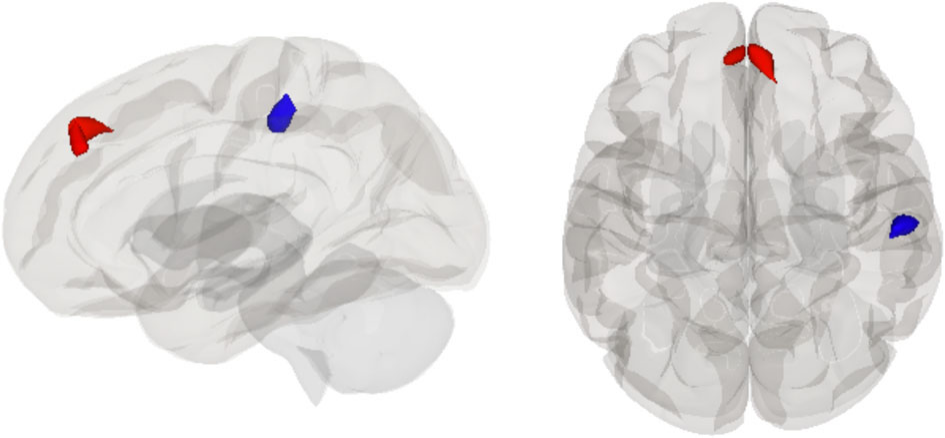
Glass brain rendering showing the independent component analysis (ICA) main effect of group on rsFC of the dorsal attention network (DAN): increased connectivity with the right superior frontal gyrus and decreased connectivity with the right supramarginal gyrus in collision players compared to contact players. rsFC, resting-state functional connectivity.

We observed a main effect of group in the DAN (see Fig. 3), such that collision sport athletes showed increased rsFC of this network with a cluster primarily in the right superior frontal gyrus and spread into the left superior frontal gyrus and paracingulate gyrus (*T* = 7.33, pFDR = 0.035) and decreased rsFC with the right supramarginal gyrus (*T* = −6.09, pFDR = 0.037). To examine this in more detail, we ran follow-up analyses. A follow-up between-subjects *t*-test contrasting the collision versus contact groups at pre-season showed increased rsFC between the DAN and a cluster primarily in the right superior frontal gyrus (*T* = 6.43, pFDR = 0.021) and decreased rsFC with a cluster in the right supramarginal gyrus (*T* = −5.15, pFDR = 0.044). No significant group differences in rsFC were observed between the DAN and other brain regions at post-season (see Table 3).

There was no significant group × time interaction.

## Discussion

Because of growing concern about the effects of exposure to RHI, this study investigated pre- versus post-season differences in cognition and brain functional integrity in contact and collision female varsity athletes. By taking a network approach, we were able to more broadly investigate rsFC changes associated with a season of play. We identified changes in DMN rsFC that showed decreased connectivity bilaterally in the superior parietal lobule and supramarginal gyrus, as well as the left superior frontal gyrus, and increased connectivity with the right frontal pole across the season, which appeared to be driven by the contact athletes. Differences in rsFC were also identified within the DAN showing increased connectivity with the right superior frontal gyrus and decreased connectivity in the right supramarginal gyrus. In contrast, we did not observe differences in attentional and executive control performance (using the ANT-I) between groups; however, there was a reduction in overall mean reaction time at post-season. This reduction may reflect a number of factors, including a test practice effect related to the ANT-I, specific training exposure associated with sport participation over the course of a season, or improvements in cognitive functioning related with cardiorespiratory fitness.^60^

Several studies have highlighted changes in the DMN after a season of play related to level of contact exposure in athletes.^15,20–22,24^ Studies have shown that non-collision sport athletes displayed a consistent DMN connectivity between pre- and post-season scans, whereas collision sport athletes have shown variability in the rsFC of the DMN throughout the season.^21,22^ Other studies have reported greater DMN connectivity in higher-contact-level athletes compared to non-contact athletes throughout the season,^20^ and in a study of football players, those who experienced a larger number of high-impact hits showed greater increases in DMN rsFC.^15^ It is striking that out of all the networks we investigated, our primary rsFC findings are consistent with the current literature, showing changes between DMN rsFC and other brain regions after a season of play, suggesting that this finding is relevant and is likely not attributable to noise.

In a comparison of pre- and post-season rsfMRI data, we observed decreased connectivity between the DMN and occipital, supramarginal, superior frontal, and superior parietal brain regions, and an increase in connectivity with the frontal pole in post-season scans. Follow-up *t*-test analyses revealed decreases in rsFC between the DMN and other brain regions after a season of play in contact sport athletes, but did not show any changes between pre- and post-season in collision sport athletes. Regions within the DMN have been associated with stability of performance.^61–63^ Specifically, decreased activity within nodes of the DMN have been associated with motor learning and improved performance.^62,64,65^ Thus, it is possible that within contact athletes, decreased rsFC between the DMN and other brain regions represents improvements in network functioning associated with visuomotor training across the season.^63,66^ A similar pattern would be anticipated in collision athletes as well, but was not observed. Thus, the absence of rsFC changes in collision athletes observed in our study may reflect the effect of greater exposure to RHIs. However, observed changes may also be attributable to additional factors, such as differences in sport-related training, the number of practices and games, as well as differences in baseline symptoms.

To further explore potential baseline differences between groups, we examined rsFC between contact and collision athletes at pre-season. We observed rsFC differences between the DAN and other brain regions that included increased rsFC with the superior frontal gyrus and decreased rsFC with the supramarginal gyrus in collision sport athletes compared to contact sport athletes. This difference may represent distinctions in specialized sport-specific attentional requirements; however, there were no group differences in altering, orienting, or executive control components of the ANT-I. There were also no significant differences in mean concussion history between contact and collision groups in our sample, and the ranges of lifetime concussions in both groups were similar (i.e., between 0 and 4 in contact players and 0 and 3 in collision players).

Other literature has shown that differences in rsFC may reflect neuroplasticity required for expertise development associated with different sport requirements^67^ and for specific relevant abilities such as motor skill learning.^68^ The demands of different sports require specialized skills of the athletes playing them, such as various adaptations to different sport surfaces and techniques. Another consideration is that our contact group was composed of athletes that played a larger variety of sports compared to the collision group, which may complicate the interpretation of the pre-season differences observed in the DAN. Additionally, no group by time interaction was observed; however, this could be attributable to small and/or unequal group sizes included in this study.

This study has several limitations, including a small sample size. However, it is notable that our sample is comparable to other neuroimaging studies examining changes in athlete functions associated with exposure to RHIs.^4,8,13,14,16,20,22,23,61^ Another limitation is the lack of accelerometry data; however, some studies have noted discrepancies between RHIs identified by accelerometry and video review,^70^ and data collected by telemetry systems have been known to overestimate linear and rotational acceleration.^71–74^ This highlights the need for the development of more accurate measurements of head impacts. Our sample also investigated differences in attention measure scores to provide an objective cognitive measure; however, we were unable to compare these scores to healthy controls, which limits our ability to interpret these scores in relation to non-athletes.

A significant variable of interest in female athletes not controlled for in this study is hormonal fluctuations caused by the natural menstrual cycle or hormonal contraceptives. Hormonal fluctuations have been shown to affect symptom endorsement after brain injuries as well as neurological findings.^17,75^ Female sex hormones, such as estrogen and progesterone, have been suggested to potentially have a neuroprotective effect in brain injuries,^76,77^ and the effect of these biological factors on RHIs should be considered in future literature.

## Conclusion

In conclusion, this study addresses important gaps in the literature and represents one of the few to examine rsFC associated with contact exposure in exclusively female athletes.^20^ We identified differences primarily in the DMN after a season of play, emphasizing the robust nature of associations between contact exposure and DMN connectivity. Specifically, our findings suggested network differences between collision and contact athletes that may be driven by pre-season differences, potentially associated with inherent differences in sport requirements. These findings highlight the complexity of examining changes directly associated with RHIs and emphasize the need for considering multiple factors (e.g., pre-season and sport differences) that might be associated with cognitive and neural changes observed over the course of a season, beyond just the suspected effects of RHIs.

## Supplementary Material

Supplemental data

## Supplementary Material

Literature Table

Expanded rs-fMRI Methods

## Abbreviations Used

ANOVA: analysis of variance
ANT-I: Attention Network Test-Interaction
DAN: dorsal attention network
DMN: default mode network
FDR: false discovery rate
FOV: field of view
FPN: frontoparietal network
ICA: independent component analysis
ISDs: individual standard deviations
MNI: Montreal Neurological Institute
MRI: magnetic resonance imaging
RHIs: repetitive head impacts
rsFC: resting-state functional connectivity
rsfMRI: resting-state functional magnetic resonance imaging
SAC: Standardized Assessment of Concussion
SCAT5: Sport Concussion Assessment Tool 5
SD: standard deviation
SMN: sensorimotor network
SN: salience network
TE: echo time
TR: repetition time
