# Supplementary material for: Altered Brain Functional Connectivity in Female Athletes Over the Course of a Season of Collision or Contact Sports
Source: Neurotrauma Rep. 2022 Sep 19;3(1):377–87. doi: 10.1089/neur.2022.0010 (PMC9531888; doi:10.1089/neur.2022.0010)
Supplement: Supplemental data [file Suppl_Data.docx]

Literature table.

| Lead Author and Study Year | PMID | Modality | Sample size (M:F) | Age: mean | Sport | Level of Competition |
| --- | --- | --- | --- | --- | --- | --- |
| Abbas et al., 2014 | 25242171 | Seed-based fMRI of DMN | 32 (32:0) | Collision: 22 (16.7 yrs, range: 14-18 yrs)  Non-contact: 10 (16.7 yrs, range: 14-18 yrs) | Football, Tennis, Cross-country, Golf, Baseball,  Swimming | High School |
| **Aim of Study:** To examine changes in the DMN after repetitive subconcussive mild traumatic brain injury.  **Concussion Criteria:** NR; **Diagnosis:** Athletic trainer, Team physician; **Post- Concussion Symptoms**: NR  **Accelerometry, neuropsychological or psychological data:** Head impact telemetry for football.  (collected but not used or reported).  **Neuroimaging Acquisition, Post-processing, & Analysis:** 3T General Electric Signa HDx, 16-channel brain array. Gradient-echo echo-planar sequence, scan length 5 min 30 sec; repetition time (TR) 1500 ms; echo time (TE) 26 ms; flip angle 35°; 34 slices at 3.8 mm; field of view 20 cm; 64 x 64 acquisition, resulting in 3.125mm x 3.125mm in-plane resolution). T1-weighted anatomical, three-dimensional spoiled gradient-recalled echo sequence (TR 5.768 ms; TE 2.032 ms; flip angle 73°; 0.9375mm· 0.9375mm· 1 mm). Analysis of Functional NeuroImages and FMRIB Software Library: preprocessing pipeline implemented where localized white matter regressors were extracted and used to implement a reduced version of the ANATICOR, which results in minimal sensitivity to motion and dependence of correlation results on censoring. Volumes associated with movement of 0.4mm or greater were censored from the regression. ROI-based correlational Analyses: Automated Anatomical Labeling atlas using the labels of Tzourio-Mazoyer divided brain into 116 ROIs. A seed region was defined by a 12mm radius spherical ROI placed at the posterior cingulate/precuneus (Talairach -5,49,40). Bivariate correlations were used as a reflection of connection between ROI pairs. Each of the 116 ROIs were considered as possible connections to the seed ROI. Non-contact athletes scanned at baseline and follow up. Collision athletes scanned 3-6 times at pre-season, at least one in-season, and post-season. ﻿For each session, ROI analyses were performed with a GLM to determine significant rsfMRI DMN connections at the individual level. Changes in the number DMN connections across sessions were evaluated in the collision-sport athletes ﻿for possible relationship to exposure to collision events by comparing each session relative to its preceding session, and by comparing each session relative to the Pre-Season session. Changes in DMN connections across consecutive sessions were evaluated using a combinatorial analysis  **Primary Neuroimaging Findings:** Baseline and follow-up sessions of non-collision-sport controls had no significantly different DMN connections between the two sessions. Collision-sport athletes showed higher numbers of significant DMN connections than non-collision-sport athletes for Pre-Season, Month-2, Month-3, and Post-Season sessions, but lower or comparable number of DMN connections for Month-1 and Month-4 sessions. At Pre-Season, Month-2, Month-3, and Post-Season, collision-sport athletes exhibited a higher (but not necessarily significantly higher) median number of DMN connections than the non-collision-sport controls. Conversely, collision-sport athletes exhibited a lower (but not necessarily significantly lower) number of DMN connections relative to their non- collision peers at Month-1 and Month-4. **Secondary Neuroimaging Findings:** The number of DMN connections exhibited greater variability across session for the collision-sport athletes. | | | | | | |
| Lead Author and Study Year | PMID | Modality | Sample size (M:F) | Age: mean | Sport | Level of Competition |
| Abbas et al., 2015 | 25649781 | Seed-based fMRI of DMN | 10  (10:0) | 17 yrs, range:16-18 yrs | Football | Highschool |
| **Aim of Study:** To examine changes in DMN connectivity for clinically asymptomatic high school football athletes  **Concussion Criteria:** NR; **Diagnosis:** Athletic trainer, Team physician; **Post- Concussion Symptoms**: NR  **Accelerometry, neuropsychological or psychological data:** NR  **Neuroimaging Acquisition, Post-processing, & Analysis:** 3T Signa HDx (General Electric), 16- channel brain array. Gradient-echo echo-planar sequence (scan length 9 min 46 sec; TR 2,000 msec; TE 26 msec; flip angle 35°; 34 slices at 3.8 mm; FOV 20 cm; 64 × 64 acquisition). T1-weighted anatomical was acquired for registration purposes (1 mm isotropic resolution). Data were preprocessed using a pipeline based on Jo et al. (2013). Correlational analysis was performed on a ROI basis. The Automatic Anatomical Labeling (AAL) atlas divided the brain into 116 ROIs. A seed region was defined by a 12 mm radius spherical ROI placed at posterior cingulate/precuneus (Talairach –5,49,40). Connectivity to this node was further analyzed as a proxy to DMN connectivity. Bivariate correlations were used as a reflection of connection between ROI pairs. A GLM was performed to determine significant rsfMRI DMN connections at the individual level. ﻿Athletes participated in nine imaging sessions; one prior to the start of contact practices (Pre-Season), two during the competition schedule (In-Season; completed within 48 hours of a game or practice), and six after the end of season (Post-Season). In-Season sessions were grouped into two month-long windows (Month-1 and Month-2). Post-Season sessions, grouped into six month-long windows (Post-1 to Post-6). During the five months of Post-Season scanning, all football athletes played another sport  **Primary Neuroimaging Findings:** The number of DMN connections exhibited marked variability across session for collision-sport athletes. DMN connections observed during the in-season scans were significantly reduced at Month-1, and significantly increased at Month-2. Post-season DMN connections were significantly reduced at all sessions except the December measurement.  **Secondary Neuroimaging Findings:** NR | | | | | | |
| Lead Author and Study Year | PMID | Modality | Sample size (M:F) | Age: mean | Sport | Level of Competition |
| Bari et al., 2019 | 29802602 | MR spectroscopy | 90 (54:36) | male collision: 40 (16.4 yrs, range: 15-18)  female collision: 23 (15.9 yrs, range: 14-17 yrs)  male non-collision: 14 (16.21 yrs, range; 15-18 yrs)  female non-collision: 13 (16.07 yrs, range: 14-18 yrs) | Football, Soccer, Cross-Country, Swimming, Track and Field, Tennis, Basketball, Softball | High School |
| **Aim of Study:** To quantify the relationship between metabolic changes and head acceleration event characteristics in high school athletes.  **Concussion Criteria:** NR; **Diagnosis:** Athletic trainer, Team physician; **Post- Concussion Symptoms**: NR  **Accelerometry, neuropsychological or psychological data:** ﻿ ﻿xPatch sensors from X2 Biosystems worn at practice and games.  **Neuroimaging Acquisition, Post-processing, & Analysis:** 3 T General Electric Signa HDx (Waukesha, WI), 16-channel brain array. Single-voxel MR spectra were acquired using the PRESS (Point RESolved Spectroscopy) pulse sequence (TR/TE = 1,500/30 ms, 128 av- erages, 2.0 × 2.0 × 2.0 cm3). A high-resolution T1-weighted anatomical scan was acquired for registration and tissue segmentation purposes using 3D spoiled gradient recalled echo (SPGR) sequence (TR/TE = 5.7/1.976 ms, flip angle = 73°, 1 mm isotropic resolution). Spectra were obtained specifically from the left dorsolateral prefrontal cortex (DLPFC) and dominant primary motor cortex (M1). ﻿﻿Tissue water reference concentrations as reported by TARQUIN were used. The values from TARQUIN were corrected for partial volume effects and for metabolite and water T1 and T2 relaxation effects—using ﻿AFNI and FSL. Collision athletes underwent five imaging sessions: one prior to participation in collision activities (Pre), one each during the first and second halves of the competition season, and two following the cessation of collision activities at intervals of 4–8 weeks (Post1) and 20–24 weeks (Post2). Non-collision athletes underwent two MRI scanning sessions, 5 to 18 weeks apart within their training/ competition seasons.  **Primary Neuroimaging Findings: ﻿** Male and female CSA pools exhibited alterations in metabolite concentration and/or metabolite ratio across sessions. In M1, the female CSA (i.e., soccer) pool exhibited a statistically ﻿significant increase in absolute concentration of M1 Glx at Post1 relative to Pre, accompanied by a statistically significant increase in the ratio [Glx]:[tCr] at In2 and Post1 relative to Pre. In DLPFC, the male CSA (i.e., football) pool exhibited a statistically significant decrease in DLPFC [Glx] at In2, relative to each of Pre, In1 and Post2. This group also exhibited a statistically significant increase in the ratio of DLPFC [tCho] to DLPFC [tCr] (i.e., [tCho]:[tCr]) at In2 relative to each of Pre, In1 and Post2. ﻿Changes across session within CSA that did not result in group-level differences with the NCA stability measurements motivated assessment whether the across-session changes for CSA were primarily confined to a sub-population of athletes who had experienced greater levels of mechanical loading. Nearly all conducted regressions evidenced their best goodness-of-fit (R2)with aPTA when optimized for Th = 50g. At this threshold, the regression for male CSA athletes at In2 of DLPFC [tCho]:[tCr] against HAE measures achieved a mean F-statistic across iterations corresponding to an uncorrected significance level of p <0.05.  **Secondary Neuroimaging Findings:** NR | | | | | | |
| Lead Author and Study Year | PMID | Modality | Sample size (M:F) | Age: mean | Sport | Level of Competition |
| Breedlove et al., 2012 | 22381736 | Task-based fMRI | 24  (24:0) | 17 yrs, range: 15-18 yrs | Football | Highschool |
| **Aim of Study:** To examine how the head collision histories of the athletes correlate with the observed changes in their neurophysiology.  **Concussion Criteria:** NR; **Diagnosis:** Clinician; **Post- Concussion Symptoms**: Clinically-observed impairment (COI) and ﻿functionally-observed impairment (FOI)— detected using neuropsychological testing and fMRI. Probed 6 components of cognitive function: attention span, working memory, sustained and selective attention time, response variability, non-verbal problem solving, and reaction time.  **Accelerometry, neuropsychological or psychological data:** Head Impact Telemetry  **Neuroimaging Acquisition, Post-processing, & Analysis:** 3T General Electric Signa HDx,16-channel brain array. Whole-brain high-resolution images (3D-FSPGR; 1mm isotropic resolution) were acquired, including the cerebellum. Three functional runs were conducted of a visual working memory (N-back) paradigm, using gradient-echo echo planar imaging (TR/TE = 1500/26 msec; matrix = 64 x 64; FOV= 20 cm; 34 slices; 3.8mm thickness; 117 volumes). In each run subjects performed one block (15 presentations, 3-sec interval, 5 targets per block) each of 0-, 1-, and 2-back tasks for single letters. Subjects responded by dominant index finger via fiberoptic button box. The order of the task blocks in the three runs was counter-balanced within each session, and across assessments. Using AFNI, working memory task conditions, were compared. 116 anatomical regions of interest (ROIs) were determined. A pre-season fMRI session was conducted before the start of contact practices. Follow-up tests were performed within 72 hours of contact activity in season 1 and within 48 hours for season 2. Individual with a diagnosed concussion is COI+/FOI+ (all of the individuals diagnosed with a concussion demonstrated functional impairment). Individuals with no diagnosed concussion and no functional impairment were COI-/FOI-. Individuals were defined as being COI-/FOI+ if they had no observable signs of concussion but nevertheless showed a statistically significant reduction in at least one of their visual composite score or verbal composite score categories for ImPACT.  **Primary Neuroimaging Findings:** Of the 116 regions of interest (ROIs), 33 yielded significant regressions relating changes in fMRI to the blows sustained by the COI-/FOI- Group. 39 ROIs yielded significant regressions relating changes in fMRI to the blows sustained by the COI+/FOI+ group. The spatial distributions of the ROIs in the COI+/FOI+ and COI-/FOI- groups for which a significant regression was obtained indicate distinct regions of correlation. While some overlap exists in the frontal, cerebellar, and basal ganglia regions, the COI-/FOI- group exhibited a concentration of correlations in the upper parietal and occipital regions. In contrast, the COI+/FOI+ group exhibited an additional concentration of correlations in the temporal and lower parietal regions.  **Secondary Neuroimaging Findings:** There was no significant difference in the median peak linear acceleration for any of the three groups, however, a significant difference in the median total number of blows was found. Subsequent pairwise tests indicated that the COI-/FOI+ group sustained more blows than the COI-/FOI- group. The number of side blows sustained by each of the groups was significantly different and pairwise tests indicated the COI-/FOI+ group sustained significantly more side blows than the COI+/FOI+ group. Scatterplots displaying a two- dimensional projection of recorded impact location and peak linear acceleration illustrated that the members of the COI-/ FOI+ group sustained a majority of their blows to the top-front region of the helmet, especially those above 80 G. | | | | | | |
| Lead Author and Study Year | PMID | Modality | Sample size (M:F) | Age: mean | Sport | Level of Competition |
| Churchill et al., 2017 | 28878729 | fMRI & DTI & Single-voxel spectroscopy | 65  (32:33) | Collision: 23 (21.3 ±1.9 yrs)  Contact control: 22 (20.3 ± 1.5 yrs)  Non-contact control: 20 (20.0 ± 1.7 yrs) | Rugby, Ice hockey, Lacrosse, Football, Soccer, Field hockey, Basketball, Water polo, Volleyball | University |
| **Aim of Study:** To evaluate multiple aspects of brain physiology in three groups of athletes participating.  **Concussion Criteria:** SCAT3 at pre-season; **Diagnosis:** In clinic; **Post- Concussion Symptoms**: NR  **Accelerometry, neuropsychological or psychological data:** NR  **Neuroimaging Acquisition, Post-processing, & Analysis:** 3T Magnetom Skyra, Siemens, standard 20-channel head receiver coil. ﻿T1-weighted MPRAGE was obtained, with field of view (FOV) = 24 cm × 24 cm, 240 × 240 × 192 acquisition matrix, 0.9 mm isotropic voxels, bandwidth = 250 Hz/Pixel, inversion time (TI)/echo time (TE)/repetition time (TR) = 850/2.63/2,000 ms, and flip angle = 8^o^. FLAIR was obtained with FOV = 22 cm × 18.6 cm, 256 × 196 acquisition matrix, 1.1 mm × 0.9 mm × 3.0 mm voxels, TI/TE/TR = 2,200/96/9,000 ms. Susceptibility-weighted imaging was also acquired with 220 × 192 FOV, 0.6 mm × 0.6 mm × 1.2 mm voxels. TE/TR = 20/28 ms, flip angle = 15°, 384 × 307 with encoding gap of 0.2 mm. ﻿Diffusion-weighted imaging was acquired with 30 encoding directions (b = 700 s/mm2, FOV = 24 cm × 24 cm, 120 × 120 acquisition matrix, 66 axial slices, 2 mm isotropic voxels, TE/TR = 83/7,800 ms, bandwidth = 1,736 Hz/Px). The FSL1 eddy_correct protocol was used to perform simultaneous correction of eddy currents and rigid-body motion correction, bet was used to mask out non-brain voxels, and dtifit was used to calculate voxelwise measures of fractional anisotropy (FA) and mean diffusivity (MD). The individual subject FA and MD maps were co-registered to a common template space, based on the FSL FDT protocol. Multi-slice T2*-weighted echo planar imaging (FOV = 20 cm × 20 cm, 64 × 64 matrix, 32 slices, 3.125 mm × 3.125 mm × 4.5 mm voxels, TE/TR = 30/2,000 ms, flip angle = 70°, and oblique axial interleaved), producing a time series of 194 images. Analysis of Functional Neuroimages (AFNI) and customized algorithms were used for processing Global FC (Gconn) was estimated for each voxel. Gconn was measured as the mean of all (positive) connectivity values, providing a voxelwise measure of total integrative function. Subsequent analyses compared the Gconn brain maps between the different sport groups. ﻿Single-voxel 1H spectroscopy data were acquired for two regions of interest, placed on left- and right-hand motor knobs. This was obtained via stimulated echo acquisition mode (STEAM) for 2 cm isotropic voxels (TM/TE/TR = 10/30/2,000 ms; bandwidth = 1,200 Hz; FA = 40°; 100 acquisitions; 1,024 points). Regions were placed on an AC-PC-oriented axial slice. Processing and analysis were conducted using the TARQUIN software package5with default preprocessing parameter settings for STEAM. N-acetyl aspartate (NAA), choline (Cho), creatine (Cr), and myo-inositol (Ins) were analyzed. All six unique pairwise ratios of the different metabolites were compared between sport groups. ﻿Mean-centered task PLS was used to identify patterns of brain voxels (FA, MD, and Gconn) or metabolite ratios that show covariation across the three sport groups (non-contact, contact, and collision).  **Primary Neuroimaging Findings:** Collision sports had higher FA than non- contact and contact sports, while non-contact and contact groups were not significantly different. Collision sports had lower MD than non-contact and contact sports while non-contact and contact were not significantly different. Gconn was highest for non-contact sports, intermediate for contact sports, and lowest collision sports. However, only collision and non-contact sports were significantly different after correcting for multiple comparisons, whereas contact sports could not be distinguished from either non-contact or collision sports. NAA/Cr ratios, showed progressive effects, with the highest values for non-contact sports, intermediate values for contact sports, and lowest values for collisions sports. Only the difference in NAA/Cr values between non-contact and collision sports was significant after correcting for multiple comparisons, while contact sports could not be distinguished from either non-contact or collision sports.  **Secondary Neuroimaging Findings:** There were no significant between-group differences for sex and prior concussion history. Pre-season symptom scores, total symptom severity was not significantly different between groups. Somatic complaints showed a significant difference between groups, with higher scores for collision sports compared to non-contact sports, although the effect was non-significant after adjusting for multiple comparisons. Cognitive and balance scores also showed no significant differences between groups. ﻿Combining all sport groups, FA within brain regions implicated in contact exposure and Gconn showed significant effects of concussion history, whereas MD and NAA/Cr ratio did not. | | | | | | |
| Lead Author and Study Year | PMID | Modality | Sample size (M:F) | Age: mean | Sport | Level of Competition |
| Johnson et al., 2014 | 25010992 | Seed-based fMRI of DMN | 24  (8:16) | Hx mTBI: 12 (20.5 yrs, range:19-23 yrs)  No Hx mTBI: 12 (19.8yrs, range: 18-22 yrs) | Rugby | University |
| **Aim of Study:** **﻿**To investigate the acute effects that subconcussive head trauma may have on the DMN.  **Concussion Criteria:** No participants reported concussive symptoms at scanning and no players were diagnosed with a concussion on the field; **Diagnosis:** Athletic trainer; **Post- Concussion Symptoms**: NR  **Accelerometry, neuropsychological or psychological data:** NR  **Neuroimaging Acquisition, Post-processing, & Analysis:** 3T Siemens Trio,12-channel head coil. Two- dimensional BOLD echo planar rsfMRI sequence were acquired in the axial plane parallel to the anterior and posterior commissure axis covering the entire brain (3.0 x 3.0x 3.0mm resolution, TR= 2490ms, TE=24ms, iPAT=none, EPI factor =74, echo spacing= 0.48ms, NSA= 1, acquisition time= 5:04). Three-dimensional isotropic T1 weighted magnetization prepared rapid gradient echo anatomical images were acquired in the sagittal plane parallel with the longitudinal fissure covering the entire brain (1mm·1mm·1mm resolution, TE= 3.46 ms, TR=2300 ms, TI =900ms, flip angle =9°, 160 slices, iPAT=none, NSA= 1). Seed-based correlation analysis of the DMN was performed. Statistical Parametric Mapping (SPM) version 8 in conjunction with FC (CONN) toolbox were used for preprocessing and analysis. Seed ﻿ROIs of the DMN included dorsal frontal cortex, anterior prefrontal cortex, orbitofrontal cortex, medial prefrontal cortex, ventral posterior cingulate cortex, posterior cingulate cortex, precuneus, angular gyrus, retrosplenial cortex, and supramarginal gyrus. ﻿Pre-game scanning was performed 24 h prior to a full contact game. Post-game scanning was performed within 24 h of the end of that game. No sports-related subconcussive impacts were sustained between pre-game scanning and the actual game, as no contact practices were scheduled in the interim.  **Primary Neuroimaging Findings:** Pre-game/post-game differences in all subjects showed increased connectivity between left and right orbitofrontal cortices and the left supramarginal gyrus and decreased connectivity between the right retrosplenial cingulate cortex and the right dorsal posterior cingulate cortex. Hx mTBI demonstrated only reductions in FC following exposure to subconcussive head trauma. There was decreased connectivity from the left anterior prefrontal cortex and left right retrosplenial cingulate cortex, from the left inferior temporal gyrus to medial prefrontal cortex and between right ventral posterior cingulate cortex and left fusiform gyrus. No Hx mTBI group exhibited only increased connectivity from pre-game to post-game. The left supramarginal gyrus increased FC to the left and right orbitofrontal cortices and between left retrosplenial cingulate cortex and right fusiform gyrus and between the left fusiform gyrus and medial prefrontal cortex. Hx mTBI cohort displayed significant decreases in FC, compared with the No Hx mTBI subgroup. ﻿Pre-game scans showed reduced FC between the left dorsal frontal cortex the left dorsal posterior cingulate cortex, right dorsal posterior cingulate cortex, and precuneus. Pre-game decreases were seen from the left supra- marginal gyrus to the left and right inferior temporal gyrus. Post-game analysis between subgroups revealed reduced connectivity from the left supramarginal gyrus to six other DMN ROIs: left and right anterior prefrontal cortices; left and right dorsal anterior cingulate cortices; right dorsal frontal cortex; and left inferior temporal gyrus.  **Secondary Neuroimaging Findings:** NR | | | | | | |
| Lead Author and Study Year | PMID | Modality | Sample size (M:F) | Age: mean | Sport | Level of Competition |
| Koerte et al., 2016 | 26286826 | Structural MRI | 30  (30:0) | Contact: 15 (49.3 ± 5.1yrs)  Non-contact:15 (49.6 ± 6.4 yrs) | Soccer, Running, Table tennis, Ballroom dancing | Former professional athletes |
| **Aim of Study: ﻿**To evaluate cortical thickness in former professional soccer players.  **Concussion Criteria:** NR; **Diagnosis:** NR; **Post- Concussion Symptoms**: NR  **Accelerometry, neuropsychological or psychological data:** Neuropsychological tests: TMT, ROCF, BIS, BESS. Lifetime-estimate of headings  **Neuroimaging Acquisition, Post-processing, & Analysis:** 3T MR Magnetom Verio, Siemens, 32-channel head coil array. T1-weighted 3D magnetization prepared rapid- acquisition gradient echo (MP-RAGE) acquired in a sagittal orientation and motion insensitive 3D T2-weighted BLADE sequence acquired in a transversal orientation. Imaging parameters were as follows: MP-RAGE: TR = 1800 ms, TE = 3.06 ms, FOV = 256 mm, voxel size = 1 × 1 × 1 mm3, iPAT, acceleration factor 2; 3D T2-weighted BLADE sequence: TR = 3000 ms, TE = 400 ms, FOV = 250 mm, voxel size =1×1×1mm3, slices = 160, iPAT, acceleration factor 2. ﻿Cortical thickness analysis was then performed using FreeSurfer version 5.3.﻿ The fully automated cortical reconstruction process was applied. Cognitive and behavioral measures were correlated with lifetime-estimate of heading. Group comparison of cortical thickness was performed. ﻿The effect of lifetime estimate of headings on cortical thickness was tested within the soccer cohort.  **Primary Neuroimaging Findings:** Group comparison revealed an interaction of group and age that involved the temporal, parietal, and occipital lobes, bilaterally. Cortical thickness within the clusters showed a significantly greater decrease with age in the soccer ﻿players compared to the control group. There were no cluster with greater decline in cortical thickness in the control group. Within the soccer players, lifetime-estimate of headings correlated with cortical thickness in a cluster that involved the right hemisphere parietal and occipital lobes. There was an overlap between the cluster with a significant correlation between life-time estimate of headings and the cluster with age x group interaction in the right inferolateral-parietal, temporal, and occipital cortex. The goalkeeper was among those with the highest cortical thickness. The two soccer players with hx mTBI injury were among those with the lowest cortical thickness.  **Secondary Neuroimaging Findings:** Soccer players performed worse on the long delay recall condition. ﻿No significant correlations were found between cognitive or behavioral measures with lifetime-estimate of headings. TMT A positively correlated with cortical thickness in the right inferolateral-parietal cortex. | | | | | | |
| Lead Author and Study Year | PMID | Modality | Sample size (M:F) | Age: mean | Sport | Level of Competition |
| Manning et al., 2020 | 32554762 | fMRI & DTI | 104  (0:104) | Contact: 73 ﻿(19.95 ± 1.5yrs)  Non-contact: 31 ﻿(19.61 ± 1.87 yrs) | Rugby, Swimming, Rowing | University |
| **Aim of Study:** To longitudinally assess brain microstructure and function in female varsity athletes participating in contact and non-contact sports.  **Concussion Criteria:** SCAT3; **Diagnosis:** Sports medicine physician; **Post- Concussion Symptoms**: NR  **Accelerometry, neuropsychological or psychological data:** Head Impact accelerometer sensor bands  **Neuroimaging Acquisition, Post-processing, & Analysis:** 3T MR Prisma, Siemens, 32-channel head coil. A coronal T2-weighted turbo spin echo sequence (echo time (TE)/repetition time (TR) = 85/7640 ms, flip angle = 120°, matrix size = 320 × 256, field of view (FOV) = 220 mm × 179 mm, number of slices = 43, slice thickness = 4 mm), a sagittal T1-weighted magnetization-prepared rapid acquisition gradient echo sequence (MPRAGE) (TE/TR = 2.94/2300 ms, flip angle = 9°, matrix size = 256 × 256, FOV = 256 mm × 240 mm, number of slices = 160, slice thickness = 1.2 mm) and a gradient-recalled multi-echo 3D image (TE/TR = 10/52 ms, flip angle = 12°, matrix size = 448 × 448, FOV = 224 mm × 168 mm, Number of slices = 128, slice thickness = 1.0 mm) were acquired. 10-min rsfMRI gradient echo [echo-planar imaging](https://www.sciencedirect.com/topics/medicine-and-dentistry/echo-planar-imaging) sequence (TE/TR = 30/2500 ms, flip angle = 90°, matrix size = 80 × 80, FOV = 240 mm × 240 mm, number of slices = 45, slice thickness = 3 mm) was performed. A spin echo DTI sequence (TE/TR = 79/7200 ms, matrix size = 98 × 98, FOV = 200 mm × 200 mm, number of slices = 64, slice thickness = 2 mm, b1 = 0, b2 = 1000 s/mm2, gradient directions = 64) was used. ﻿Diffusion data were analyzed with the Functional Magnetic Resonance Imaging of the Brain (FMRIB) Software ﻿rsfMRI data were preprocessed with the fMRI Expert Analysis Tool in FSL. ﻿Independent component analysis was used to identify resting-state networks including ﻿the DMN, lateral visual network, and cerebellar. Imaging ROIs with significant differences among groups were explored further with a linear mixed- effects model within MATLAB to evaluate the main effect of participant group, time, and interaction. ﻿DTI and rsfMRI data were acquired during the in-season and off-season if the athlete did not experience a diagnosed concussion within 6 months of entry into the study.  **Primary Neuroimaging Findings:** Concussion-free contact athletes during both the in- and off- season had greater connectivity between the DMN and the posterior cingulate cortex (PCC) compared to in- and off- season non-contact. Connectivity between the lateral visual network and areas throughout the occipital lobe was increased for in- and off-season contact athletes compared to in-season non-contact athletes. There were no significant differences in functional connectivity in the cerebellar RSN after correction for multiple comparisons. ﻿No significant differences in connectivity between the PCC and DMN in rugby athletes with and without a concussion history. ﻿Significant differences across all diffusion metrics between concussion-free contact athletes and non-contact athletes (corpus callosum, cingulum, and brainstem, portions of the superior longitudinal and inferior occipital fasciculi). Significant effect of group where AD was higher in the contact athletes and MD was higher in contact athletes compared to in-season non- contact athletes in the splenium and genu of the corpus callosum. MD, AD, and RD of the body of the corpus callosum had a significant group-by-time interaction, and main effects for group and time, and were all higher in both in- and off-season for contact compared to in-season non-contact.  **Secondary Neuroimaging Findings:** FA in the brainstem was significantly associated with the SCAT immediate memory subtest scores. | | | | | | |
| Lead Author and Study Year | PMID | Modality | Sample size (M:F) | Age: mean | Sport | Level of Competition |
| Mayinger et al., 2018 | 28092023 | DTI | 20  (20:0) | Collision: 15 (﻿20.0 ± 1.0 yrs)  Non-athlete: 5 (﻿20.93 ± 1.1 yrs) | Football | University |
| **Aim of Study:** To evaluate longitudinal changes in the diffusion characteristics of brain white matter (WM) in collegiate athletes at three time points: prior to the start of the football season (T1), after one season of football (T2), followed by six months of no-contact rest (T3).  **Concussion Criteria:** SCAT2; **Diagnosis:** Athletic trainer; **Post- Concussion Symptoms**: ImPACT  **Accelerometry, neuropsychological or psychological data:** NR  **Neuroimaging Acquisition, Post-processing, & Analysis:** 3T Tim Trio system Siemens, 32-channel matrix head coil. DTI sequence parameters were TR/TE =10 s/89 ms, voxel size 2x2x2 mm, 60 diffusion directions with b = 1200 s/mm2 and 10 averages of b = 0. ﻿WM diffusion characteristics were analyzed using TBSS. ﻿Timepoint and individual specific diffusion values were obtained from the statistically significant voxel clusters. Average diffusivity values from significant voxels were plotted using GraphPad Prism. Statistically ﻿significant FA clusters were used to create an ROI. which was applied to the FA map to obtain FA values in that same ROI. Significant clusters of voxels identified in the athlete group were then applied to the control group, and diffusion values in these clusters were measured. All subjects underwent DTI at baseline/pre-season (T1), immediately post-season (T2), and six-months post-season (T3).  **Primary Neuroimaging Findings:** Significant T1 to T2 increase in FA in the left parietal lobe. After six months of rest (T2 to T3) there was a decrease in FA in this same cluster. TBSS also identified a cluster of voxels with a significant T2 to T3 decrease in FA in a slightly different voxel cluster also located in the left parietal lobe. There were no significant changes in FA from T1 to T3 for either cluster. ﻿T1 to T2 increase in trace in the brainstem and in the left temporal lobe. After six months of rest (T2 to T3), there was a decrease in trace in this cluster.  **Secondary Neuroimaging Findings:** Exposure among athletes ranged from 48 to 1850 total impacts (mean ± SD = 800 ± 515). There were no significant group mean declines in cognitive performance at any time point. | | | | | | |
| Lead Author and Study Year | PMID | Modality | Sample size (M:F) | Age: mean | Sport | Level of Competition |
| Monroe et al., 2020 | 32861790 | fMRI | 24  (24:0) | Participants: 24 (﻿20.2 ± 1.5 yrs)  Contact: 13  Non-contact: 11 | Soccer, Golf, Cross-country | University |
| **Aim of Study: ﻿**To examine the relationship between head-to-ball impact exposure and changes in functional connectivity in a core set of central autonomic regions and then to determine the relation between changes in brain and changes in behavior, specifically cognitive control.  **Concussion Criteria:** NR; **Diagnosis:** Athletic trainer; **Post- Concussion Symptoms**: NR  **Accelerometry, neuropsychological or psychological data:** Dot pattern expectancy task  **Neuroimaging Acquisition, Post-processing, & Analysis:** 3T Philips Achieva, sensitivity encoding (SENSE) 32-channel head coil. T1-weighted fluid attenuated inversion recovery (FLAIR) sequence (flip angle = 90°; matrix size = 240 × 240; number of slices = 180; slice thickness = 2 mm). Functional images were acquired using a T2-weighted EPI sequence (TE = 29 ms; TR = 2000 ms; matrix size = 64 × 64; number of slices = 51; flip angle = 71°).﻿﻿ FSLeyes was used to create ROIs. ROIs were placed in the ventral anterior insula, medial prefrontal and medial orbitofrontal cortices, amygdala and subgenual anterior cingulate cortex, periaqueductal grey, and hypothalamus. ﻿CONN functional connectivity toolbox was used to preprocess images and perform functional connectivity statistical analyses. ﻿Functional connectivity within the core central autonomic network for each participant at pre- and post-season was defined using partial correlations, which represent linear relationships between two nodes conditioned on all other connections in the network. ﻿GLM to determine whether head impact exposure (independent variable) was associated with changes in connectivity be- tween each pair of CAN nodes. ﻿A ‘brain’ matrix, representing thresholded and residualized post-season core CAN functional connectivity, and a ‘behavior’ matrix of residualized DPX scores, served as inputs to a partial least squares (PLS) correlation analysis. ﻿Athletes completed two rsfMRI scans, one before and one after the sea- son, within 2 weeks of the beginning and end of potential exposure to head-to-ball impacts  **Primary Neuroimaging Findings:** Head-to-ball impact exposure was associated with changes in FC within a subset of 40 edges between all 14 nodes of the core CAN. The left insula, left hypothalamus, and left amygdala exhibited the greatest integration within this subnetwork, meaning they were most affected by head-to-ball impact exposure. The right amygdala and left and right medial PFC were least affected. PLS revealed CAN was associated with a decreased PBI (indicating a greater reliance on reactive cognitive control strategies), but not with a change in d-prime.  **Secondary Neuroimaging Findings:** NR | | | | | | |
| Lead Author and Study Year | PMID | Modality | Sample size (M:F) | Age: mean | Sport | Level of Competition |
| Sollmann et al., 2018 | 29204342 | DTI | 25  (14:11) | Male: 14 (﻿21.7 ± 1.3 yrs)  Female: 11 (﻿19.2 ± 1.8 yrs) | Hockey | University |
| **Aim of Study:** **﻿**To identify and to characterize sex differences following exposure to repetitive subconcussive head impacts.  **Concussion Criteria:** Per the ﻿Zürich consensus statement; **Diagnosis:** Specialist physician; **Post- Concussion Symptoms**: ImPACT  **Accelerometry, neuropsychological or psychological data:** NR  **Neuroimaging Acquisition, Post-processing, & Analysis:** 3T MRI scanner, 8 channel head coil. A sequence with two averages and 60 non-colinear diffusion directions (TR/TE: 7015 ms/60 ms, b: 0 and 0.7 ms/mm2, 70 slices) was acquired using a 2.2 mm isotropic voxel size and a 100 × 100 matrix reconstructed into a 112 × 112 matrix with a resolution of 2 × 2 × 2.2 mm3. ﻿For analysis of WM diffusion properties, tract-based spatial statistics (TBSS) were carried out. ﻿The voxels that formed the skeletons were extracted for each individual scan. Pre-season data sets were subtracted from the post- season data sets, which generated skeletonized delta maps for each participant for FA, MD, AD, and RD, respectively. ﻿To identify voxel clusters with statistically significant group differences between females and males in the change in diffusion scalar measurements over the course of the play season, unpaired t-tests were performed applying the randomise command.  **Primary Neuroimaging Findings:** Differences between male and female participants in change over time. The statistically significant FA cluster primarily includes the superior longitudinal fasciculus (SLF), internal capsule (IC), and corona radiata (CR) of the right hemisphere. In the statistically significant cluster, FA values did not change significantly in male participants over the course of one season, whereas a decrease in FA in female participants was observed. The statistically significant MD cluster mainly includes the SLF, IC,﻿ CR, and the external capsule (EC) of the RH. In the significant voxel cluster, MD did not change significantly in male participants, whereas female participants demonstrated an increase in MD. Both AD and RD, values increased in female participants over the course of one season, whereas they did not in male participants.  **Secondary Neuroimaging Findings:** There were no statistically significant differences between female and male participants﻿ except for visual motor speed at post-season assessment, where male athletes demonstrated significantly improved function in visual motor speed compared to females. | | | | | | |
| Lead Author and Study Year | PMID | Modality | Sample size (M:F) | Age: mean | Sport | Level of Competition |
| Slobounov et al., 2017 | 28393012 | fMRI & DTI & CBF | 18  (18:0) | Participants: 18 (21.6 ± 1.28 yrs) | Football | University |
| **Aim of Study: ﻿**To examine the effects of repetitive collisions across a single competitive season in NCAA Football Bowl Subdivision athletes using advanced neuroimaging approaches.  **Concussion Criteria:** NR; **Diagnosis:** NR; **Post- Concussion Symptoms**: NR  **Accelerometry, neuropsychological or psychological data:** BodiTrak system from HeadHealth Network -helmet sensors worn only at practices  **Neuroimaging Acquisition, Post-processing, & Analysis:** 3T Prisma MR Siemens, 32-channel head coil. T1-weighted 1-mm3 ﻿isotropic volumetric images (3 min 31 s), with cerebrospinal fluid (CSF) suppressed, were obtained to cover the whole brain with a 3D magnetization prepared rapid acquisition gradient recalled echo (3D MPRAGE) sequence with the following parameters: TE = 1.77 ms, time of inversion (TI) = 850 ms, TR = 1700 ms, flip angle = 9°, matrix size = 320 × 260 × 176, voxel size = 1 mm× 1 mm× 1 mm, receiver bandwidth = 300 Hz/pixel, and parallel acceleration factor = 2. 10-min echo-planar: 72 contiguous 2-mm axial slices in an interleaved order, time of echo (TE) = 35.8 ms, time of repetition (TR) = 2000 ms, flip angle = 90°, voxel resolution = 2 mm× 2 mm× 2 mm, matrix size = 104 × 104, 300 total volumes acquired. Regional cerebral blood flow data were acquired using 3D ASL (6 min 2 s): 40 axial slices, TE = 15.62 ms, TR = 4600 ms, voxel resolution = 1.5 mm× 1.5 mm× 3 mm, field of view = 192 mm× 192 mm, bolus duration = 700 ms, perfusion mode set at PICORE Q2TIPS, parallel acceleration factor = 2. White matter integrity was assessed using Tract-Based Spatial Statistics (TBSS) in FSL. Two-tail paired t-tests comparing Pre and Post measures of FA, MD, RD, and AD were applied. ﻿All cortical and subcortical volumes were extracted from the T1- weighted images via the FreeSurfer standard processing pipeline. Two-tail paired t-tests were applied to assess all regional cortical and subcortical volume differences between Pre and Post measurements. AFNI software was used to generate the scripts to preprocess the rs-fMRI data. ﻿Correlation analyses of rs-fMRI time courses between the nodes of each network were performed. Comparison analyses between two sessions were performed on the transformed correlations with the seed regions. ﻿SWI data were examined for changes between Pre and Post measurements that might indicate development of leaks. Players completed both MRI scans: within one week before the athletic season began (before any contact practices began during pre-season and the regular season) and within one week after the last game of the season (post-season)  **Primary Neuroimaging Findings:** No significant changes on the cortical and subcortical regional volumes were found. ﻿No significant changes were observed when contrasting Pre and Post average connectivity within each of the 17 networks. ﻿Similarly, no significant changes were observed when contrasting the Pre and Post within-network. ﻿Seed-based whole-brain functional connectivity analyses showed significant changes from Pre to Post in FC of clusters to seed regions were only observed for three of the four seed regions (right ICC, left ICC, left hippocampus). There was a statistically significant global increase of CBF in the cortex from Pre to Post. ﻿No significant changes were observed when contrasting DTI metrics (FA, MD, AD and RD) at Pre and Post measurements.  **Secondary Neuroimaging Findings:** On a per-practice-session basis, each player received an average of 4.35 impacts meeting or exceeding 25G, with an average of 0.15 of these impacts meeting or exceeding 80G. ﻿No individual regions exhibited a statistically significant relationship between impact history metrics and measures either of CBF or functional connectivity. Greater increases in DMN connectivity over the course of the season, increase from Pre to Post in CBF, and statistically significant numbers of regions with decreased SWI signal intensity were found to be associated with athletes who experienced a broader/ higher distribution of per-practice-session impacts exceeding ≥80G. | | | | | | |
| Lead Author and Study Year | PMID | Modality | Sample size (M:F) | Age: mean | Sport | Level of Competition |
| Svaldi et al., 2020 | 30377933 | fMRI  MR spectroscopy | 21 (0:21) | ﻿Participants: 21  (15.9 ± 1.0) | Soccer | High School |
| **Aim of Study: ﻿**To model the relationship between (subconcussive) head acceleration event accumulation and cerebrovascular reactivity changes in a cohort of female high school soccer athletes.  **Concussion Criteria:** NR; **Diagnosis:** NR; **Post- Concussion Symptoms**: NR  **Accelerometry, neuropsychological or psychological data:** ﻿ ﻿xPatch sensors from X2 Biosystems worn at practice and games.  **Neuroimaging Acquisition, Post-processing, & Analysis:** **﻿**3 T General Electric Signa HDx (Waukesha, WI),16- channel brain array. Cerebrovascular reactivity was measured using a hypercapnic breath hold challenge. For each imaging session, a single blocked breath-hold FMRI run (4 breath holds, 20s duration, separated by paced breathing, hold on the exhale) was acquired in each session using a gradient- echo echo planar sequence (TR/TE = 1500/26 msec; 20 cm FOV; 64 × 64 matrix; 34 slices; 3.8 mm thickness; 117 volumes). T1-weighted anatomical scan were acquired using a 3D spoiled gradient echo sequence (TR/TE 5.758 ms/2.032 ms, flip angle = 73°, 1 mm isotropic resolution). **﻿** FMRI analyses were performed using AFNI. A processing stream adapted from afni_proc.py, including slice timing correction, motion correction, spatial smoothing, alignment to the structural scan, normalization to Talairach space, and conversion to percent signal change, was used. Additionally, the FAST automated segmentation tool in FSL was used to create a GM mask for each subject. FMRI analyses were conducted at both the whole brain level (GM only) and at the gyrus level (GM only). For gyrus level analyses, the brain was parcellated into 70 regions of interest. Athletes were imaged once before the onset of the current season’s collision activities (Pre), once each during the first half (approximately 5 weeks) of the contact season (In1) and the second half (In2), and once at an interval of 1–2 months after the end of the competition season (Post).  **Primary Neuroimaging Findings:** The mean CVR (i.e., cerebrovascular reactivity) fractional change from Pre, was found to be significantly decreased from zero at all follow up imaging sessions. The cumulative load coefficient was only found to be significantly different from zero for HAES (i.e., head acceleration events) above 50 g. At this 50 g threshold, nCPTA (i.e., normalized cumulative peak translational accelerations) explained 23.3% of the variance in CVR change from Pre. No fits or coefficients were found to be statistically-significant for the In1 (i.e.., first half of season) or Post sessions. At In2 (ie., second half of season), for all threshold values assessed in the range Th =30g 70 g, nCPTA was found to have a significant effect on CVR changes from Pre p<0.05, F-test, with the maximum variance explained found at a threshold of 50 g.  **Secondary Neuroimaging Findings:** There was no significant effect of age at any session on CVR changes from Pre. | | | | | | |
| Lead Author and Study Year | PMID | Modality | Sample size (M:F) | Age: mean | Sport | Level of Competition |
| Talavage et al., 2014 | 20883154 | Task based fMRI | 11  (11:0) | Participants: 11 (Range: 15-19 yrs) | Football | Highschool |
| **Aim of Study:** To ﻿examine neurological performance and health in the presence of head collision events in high school football players.  **Concussion Criteria:** Clinically observed impairment as diagnosed by team physician (COI+). Deviant ImPACT re- tests were said to be positive for a functionally observed impairment (FOI +); **Diagnosis:** Team physician; **Post- Concussion Symptoms**: NR  **Accelerometry, neuropsychological or psychological data:** Head Impact Telemetry  **Neuroimaging Acquisition, Post-processing, & Analysis:** 3T General Electric Signa HDx,16-channel brain array. Whole-brain high-resolution images (3D-FSPGR; 1mm isotropic resolution) were acquired, including the cerebellum. Three functional runs were conducted of a visual working memory (N-back) paradigm, using gradient-echo echo planar imaging (TR/TE = 1500/26 msec; matrix = 64 x 64; FOV= 20 cm; 34 slices; 3.8mm thickness; 117 volumes). In each run subjects per- formed one block (15 presentations, 3-sec interval, 5 targets per block) each of 0-, 1-, and 2-back tasks for single letters. Subjects responded by dominant index finger via fiberoptic button box. The order of the task blocks in the three runs was counter-balanced within each session, and across assessments. Data were analyzed using AFNI. Pre-processing included slice timing correction, motion correction, normalization to Talairach space, and 8-mm gaussian smoothing for inter-subject comparison. Final analysis for each subject was affected on concatenated data, using a general linear model approach with Gamma Variate hemodynamic response function (without derivatives). The contrast of interest is a comparison between 2-back and 1-back working memory tasks, with statistically significant activation. Changes in fMRI activation were assessed using the 116 anatomically defined regions of interest (ROIs) from MarsBaR. Cross-modality analyses were performed to assess whether subsequently observed changes in fMRI assessment of physiology were correlated with head collision events. ﻿Alterations of hemodynamic response signal amplitudes observed during in-season fMRI relative to that observed within the same subject in the pre-season assessment, was compared to the number of head-collision events measured by the HIT System in the week prior to the in-season assessment. This assessment was performed both on an anatomical ROI basis, and on a more global basis, for an aggregated ROI encompassing nearly the entirety of the frontal lobe, excluding only the precentral gyrus. A 3x2 implementation of the test was used, in which the player categories are considered the treatments, and the observation is either a decrease or a non-decrease in the in-season frontal lobe hemodynamic response signal amplitude, relative to that obtained from the pre-season assessment, evaluated on a per- subject basis. Athletes completed ImPACT and fMRI assessments at pre-season, in-season (within 48h of a game or 72h of diagnosis of concussion), and post-season (1-3months following season). ﻿Players who were diagnosed by the team physician with a concussion were deemed to be positive for clinically observed impairment and are labeled COI+. Players who exhibited deviant ImPACT retests were said to be positive for a functionally observed impairment (FOI+). ﻿Scores within the 99% confidence intervals were negative. ﻿Collision events recorded by the HIT System for each player were analyzed using a one-way analysis of variance (ANOVA).  **Primary Neuroimaging Findings:** fMRI data for COI+ /FOI + players revealed alterations in the pattern and amplitude of signal differences observed when contrasting the 2-back and 1-back memory tasks, particularly in the posterior middle and superior temporal gyri. In-season fMRI data for COI - /FOI – players showed no differences in 115 of the 116 ROIs, both on a within-player basis, and relative to the group random effects analysis. (exception: right cerebellum 3, which exhibited decreased activation in three players). At post-season, COI- /FOI - players ImPACT scores and task performance were again found to be within test/re-test limits. COI-/FOI- at all in-season assessments, significantly decreased fMRI activation in the dorsolateral prefrontal cortex and cerebellum. In particular, when the 2-back and 1-back working memory conditions were contrasted, activation in the DLPFC changed from favoring the 2-back condition, to favoring the 1-back condition. When compared with COI+ /FOI + players, the COI- /FOI + players were found to be at least as impaired (as demonstrated by both ImPACT and fMRI measures) as the known-concussed group.  **Secondary Neuroimaging Findings:** Three players were categorized as COI+ /FOI +, four as COI- /FOI-, and four as COI- /FOI +. The total number of collision events experienced by the COI- /FOI + group was significantly greater than that of any other group and exhibited more high magnitude ( > 80g) collision events directed to the top front of the helmet immediately above the DLPFC. The number of head collision events experienced in the week preceding an in-season assessment was correlated with changes in fMRI activation for the 2-back versus 1-back contrast of interest. | | | | | | |

**ANTI test administration information**

Participants completed the ANT-I, which is a 20-minute computerized test measuring aspects of attention and executive control by examining RT performance across three domains: alerting (auditory tone), orienting (visual cue), and executive control (congruent and incongruent flankers). Participants are presented with a series of five arrows located either above or below a central fixation cross and have to indicate the direction of the center arrow by pressing the "/" (right) or "z" (left) keys on a standard computer QWERTY keyboard. The test was composed of 25 practice trials and 288 test trials. The trials were presented at variable intervals, with a fixation cross appearing in the center of the screen between each trial. The intervals ranged from 400 to 1600 ms, and each trial lasted for 4450 ms. An alerting stimulus in the form of a 2000 Hz tone was played on half of the trials for 50 ms, followed by an orienting stimulus that appeared after 100 ms in the form of an asterisk on two-thirds of the trials for 50 ms. In addition, one third of trials were randomly presented with a valid spatial cue, with the asterisk located either above or below the central fixation cross, correctly signalling the upcoming position of the target stimulus. Another third of trials were presented with an invalid spatial cue, incorrectly signalling the target stimulus' upcoming position, and the remaining third of trials had no cue associated with them (see Figure A below).


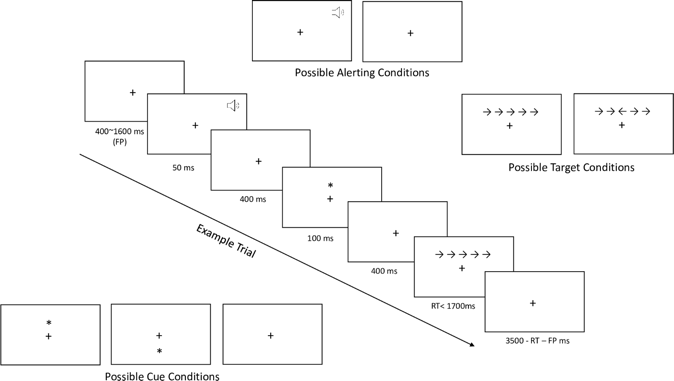


Figure A. Attention Network Test (ANT-I). Example trial: Following a 400–1600 millisecond (ms) inter-trial period, the target stimulus is preceded by an alerting tone (second top panel) and a valid orienting cue (fourth top panel). The target (center) arrow is surrounded by incongruent flankers (second bottom panel). Possible cue, target (i.e., congruent and incongruent flankers), and alerting conditions are also displayed.

**Expanded rs-fMRI methods**

Using CONN toolbox Version 18.b (Whitfield-Gabrieli & Nieto-castanon, 2012) on the Matlab Version R2019a platform, fMRI data (i.e., the second echo from the multi-echo scan) were preprocessed. The default preprocessing pipeline was used and functional data were functionally realigned and unwarped, translated by centering to (0,0,0) coordinates, slice-time corrected, scrubbed with ART-based identification for outlier scans, segmented into grey matter (GM), white matter (WM), and cerebrospinal fluid (CSF), normalized to the Montreal Neurological Institute (MNI) template MNI152, and smoothed using an 8 mm Gaussian kernel, full width at half maximum. Denoising was performed using linear regression of the following nuisance parameters: 1) Realignment parameters (n = 12): 6 realignment parameters (three translational and three rotational) as well as the first temporal derivative of each; 2) Condition effect (n = 2): constant and linear BOLD signal within each session to reduce the influence of slow trends and/or initial magnitization transience in the BOLD signal; 3) Spike regression (n = 0-15): one regressor for each outlier time-point (n-range = 0-15 across participants) in the time-series; 4) White matter signal (n = 5): 5 temporal principal components; and 5) Cerebral spinal fluid (n = 5): 5 temporal principal components. Mean global signal regression was not performed in light of the potentially detrimental effects which have been debated in recent literature (Gotts et al., 2013, 2020; Murphy et al., 2009; Saad et al., 2012; Spreng et al., 2019). Finally, data were linearly de-trended and default band-pass filtered (0.008-0.09 Hz). Quality assurance plots were produced to indicate number of valid scans, max and mean motion, and max and mean global signal change for each subject.

**References**

Gotts, S. J., Saad, Z. S., Jo, H. J., Wallace, G. L., Cox, R. W., & Martin, A. (2013). The perils of global signal regression for group comparisons: A case study of Autism Spectrum Disorders. *Frontiers in Human Neuroscience*, *JUL*. <https://doi.org/10.3389/fnhum.2013.00356>

Murphy, K., Birn, R. M., Handwerker, D. a, Jones, T. B., & Bandettini, P. a. (2009). The impact of global signal regression on resting state correlations: are anti-correlated networks introduced? *NeuroImage*, *44*(3), 893–905. <https://doi.org/10.1016/j.neuroimage.2008.09.036>

Saad, Z. S., Gotts, S. J., Murphy, K., Chen, G., Jo, H. J., Martin, A., & Cox, R. W. (2012). Trouble at Rest: How Correlation Patterns and Group Differences Become Distorted After Global Signal Regression. *Brain Connectivity*, *2*(1), 25–32. <https://doi.org/10.1089/brain.2012.0080>

Spreng, R. N., Fernández-Cabello, S., Turner, G. R., & Stevens, W. D. (2019). Take a deep breath: Multiecho fMRI denoising effectively removes head motion artifacts, obviating the need for global signal regression. In *Proceedings of the National Academy of Sciences of the United States of America* (Vol. 116, Issue 39, pp. 19241–19242). National Academy of Sciences. <https://doi.org/10.1073/pnas.1909848116>

Whitfield-Gabrieli, S., & Nieto-castanon, A. (2012). Conn: a functional connectivity toolbox for correlated and anticorrelated brain networks. *Brain Connectivity*, *2*(3), 125–141. https://doi.org/10.1089/brain.2012.0073
